# Relationship between Body Posture Assessed by Dynamic Baropodometry and Dental Occlusion in Patients with and without Dental Pathology

**DOI:** 10.3390/s24061921

**Published:** 2024-03-17

**Authors:** Isabel Carda-Navarro, Lidia Lacort-Collado, Nadia Fernández-Ehrling, Alicia Lanuza-Garcia, Javier Ferrer-Torregrosa, Clara Guinot-Barona

**Affiliations:** 1 Dentistry Department, Faculty of Medicine and Health Sciences, Catholic University of Valencia San Vicente Mártir, 46001 Valencia, Spain; isabel.carda.18@mail.ucv.es (I.C.-N.); alicia.lanuza@ucv.es (A.L.-G.); clara.guinot@ucv.es (C.G.-B.); 2 Podiatry Department, Faculty of Medicine and Health Sciences, Catholic University of Valencia San Vicente Mártir, 46001 Valencia, Spain; lidia.lacort@ucv.es (L.L.-C.); nadia.fernandez@ucv.es (N.F.-E.)

**Keywords:** body posture, dental occlusion, baropodometry, malocclusions, center of pressures

## Abstract

Body biomechanics and dental occlusion are related, but this interaction is not fully elucidated. The aim of this study was to investigate the association between body posture and occlusion in patients with and without dental pathology. A cross-sectional study was carried out with 29 patients divided into a control group and a group with pathology (malocclusions). Body posture was evaluated by dynamic baropodometry, analyzing parameters such as the line of gait and the anteroposterior and lateral position of the center of pressure (CoP). Occlusion was classified radiographically according to the sagittal skeletal relationship. Results showed significant differences in mean position phase line between groups (*p* = 0.01–0.02), with means of 115.85 ± 16.98 mm vs. 95.74 ± 24.47 mm (left side) and 109.03 ± 18.03 mm vs. 91.23 ± 20.80 mm (right side) for controls and pathologies, respectively. The effect size was large (Cohen’s d 0.97 and 0.92). There were no differences in the anteroposterior (*p* = 0.38) or lateral (*p* = 0.78) position of the CoP. In gait analysis, significant differences were observed in left (548.89 ± 127.50 N vs. 360.15 ± 125.78 N, *p* < 0.001) and right (535.71 ± 131.57 N vs. 342.70 ± 108.40 N, *p* < 0.001) maximum heel strength between groups. The results suggest an association between body posture and occlusion, although further studies are needed to confirm this relationship. An integrated postural and occlusal approach could optimize the diagnosis and treatment of dental patients.

## 1. Introduction

Biomechanics studies the internal and external forces acting on the human body and the movement produced by these forces. In the context of gait, biomechanics analyzes parameters such as velocity, acceleration, ground reaction forces, momentum, and joint power. The Zebris pressure platform (Zebris Medical GmbH, Weitnau, Germany) [1,2,3] allows the assessment of dynamic biomechanics during gait by recording the distribution of plantar pressures using sensors.

Zebris provides accurate information on several relevant biomechanical parameters, such as the center of pressure (CoP) and its displacements in the anteroposterior and mediolateral axes. The analysis of these variables in gait is essential to understanding the forces involved, postural control, and gait patterns of individuals. The Zebris platform represents an invaluable tool in clinical biomechanical research [1], allowing the detection of subtle alterations in movement dynamics that could be related to various pathologies, such as scoliosis [2], cerebral palsy [3], or Parkinson’s disease [4]. Body posture, which involves the position of the body in relation to gravity, can be altered due to various factors, such as hyperlordosis, hyperkyphosis, or deviations of the pelvis [5,6]. Postural assessment detects these alterations by means of methods such as visual observation or baropodometry, which analyzes the distribution of pressures in the feet during standing [7,8,9,10,11]. Abnormal plantar pressure may be associated with deformities in body alignment [12]. A possible association between body posture and dental occlusion has been suggested [13], as well as with problems in the stomatognathic system, although more research is needed to confirm these relationships [14,15,16]. Compensatory skeletal or muscular adaptations may be influenced by jaw position, unilateral mastication, malocclusions, or temporomandibular disorders.

Posturological analysis evaluates parameters such as spinal alignment and balance between the feet [17,18,19,20]. The “butterfly diagram” [21,22,23,24] is used to visualize body weight distribution during gait [25,26]. Usually, in biomechanical studies, the study of the Foot Posture Index is used [27,28,29], but in this study, it is performed by gait analysis with the butterfly diagram analyzing the relationship with body posture and its different speeds during gait [30].

Skeletal class is a crucial concept in orthodontics and dentistry, referring to the categorization of an individual’s facial and dental structure [31,32]. Three main skeletal classes are distinguished (Class I, II, and III) [33] describing the relationships between the maxilla and mandible. Class I indicates a normal relationship, whereas Class II [34] indicates a receded position of the mandible, generating an overbite. In contrast, Class III [31,35] implies a forward mandible, resulting in a crossbite. The classification essentially guides the evaluation of malocclusions, allowing dental health professionals to determine appropriate treatment strategies, whether through the use of braces, orthopedic appliances, or other orthodontic procedures. This information is also valuable in orthognathic surgery to address more severe cases of malocclusion. In this context, it is proposed to unify samples into two groups based on the skeletal classes, pathology and normal, thus facilitating a more specific classification to see if there is any relation to the posturology of the foot.

The treatment of postural and occlusal disorders requires a multidisciplinary approach, including physiotherapy, neuro-occlusal rehabilitation, or orthodontics. The goal is to restore musculoskeletal balance and a bilateral physiological masticatory pattern to prevent or correct possible postural compensations.

The primary aim of this research is to delve into the intricate relationship between body posture and dental occlusion. Conducting both postural analysis and functional assessment of occlusion yields pertinent insights crucial for accurate diagnosis and comprehensive treatment across multiple disciplines. Our hypothesis posits a correlation between gait irregularities and resultant skeletal class alterations in patients.

## 2. Materials and Methods

### 2.1. Study Design

A cross-sectional study was performed including two groups of patients (i.e., patients with dental pathology vs. non-pathological). This study was approved by the Research Ethics Committee of the Catholic University of Valencia (UCV/2021-2022/200) in accordance with the ethical guidelines of the Declaration of Helsinki [36]. In addition, the design and progression of participants throughout the trial were performed according to the STROBE guidelines [37] (Supplementary Materials). All patients were recruited from the same clinic. Prior to the testing procedures, all patients gave written informed consent [36,37].

### 2.2. Participants

**Figure 1:**
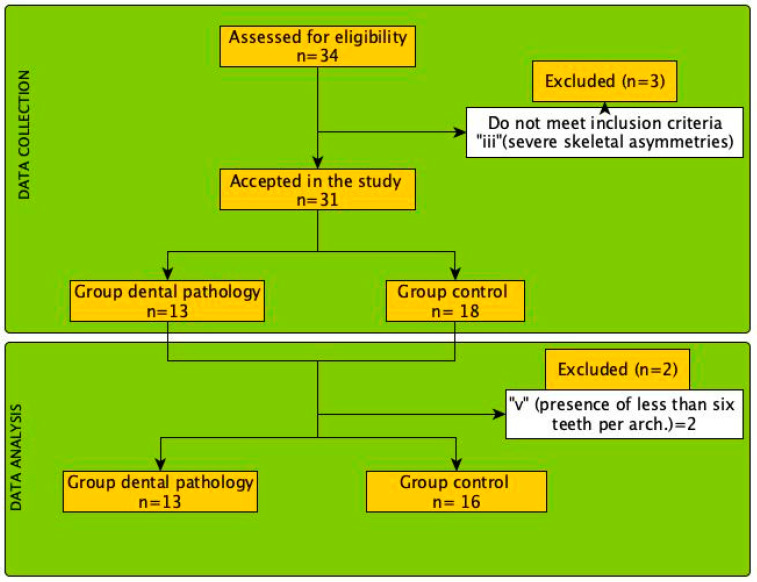
Flow diagram of the selection process and analysis of the participants included in the present study.

A total of 29 patients were divided into two groups, depending on whether they had dental pathology. Participants’ measured characteristics are presented in Table 1. The inclusion criteria were: (i) permanent dentition, (ii) absence of previous orthodontic treatment, (iii) absence of systemic pathology, and (iv) good general health. The exclusion criteria were: (i) having received orthodontic treatment, (ii) presenting craniofacial syndromes, (iii) severe skeletal asymmetries, (iv) history of maxillofacial surgery, and (v) presence of less than six teeth per arch (see Figure 1).

### 2.3. Evaluation

**Figure 2:**
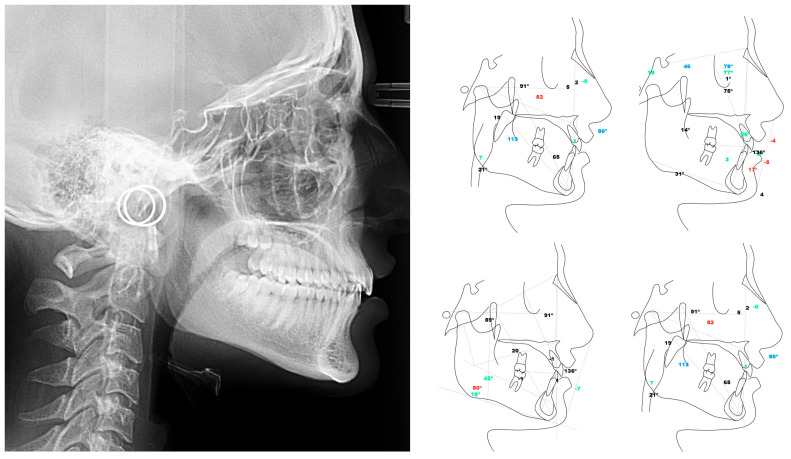
Radiography to measure skeletal class and to classify patients into pathological and non-pathological.

The evaluations were carried out at the UCV University Clinics. During the first visit, an intraoral clinical examination was carried out to identify possible dental and skeletal alterations. Subsequently, a lateral skull teleradiography was performed with a cephalostat to standardize the patient’s position. This radiograph was used to classify the class in the sagittal plane and to determine whether or not there was dental pathology (see Figure 2). Class 1 was classified as non-pathological (Group control) and Class 2 and 3 as dental pathologies. All measurements were performed with NemoCeph NX software 1.0 (Nemotec, Madrid, Spain).

### 2.4. CoP Analysis

**Figure 3:**
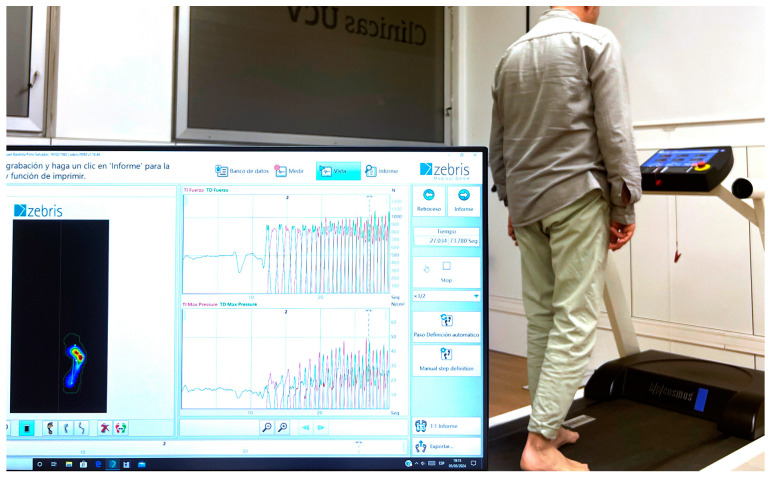
Zebris FDM platform for gait and posture analysis.

**Figure 4:**
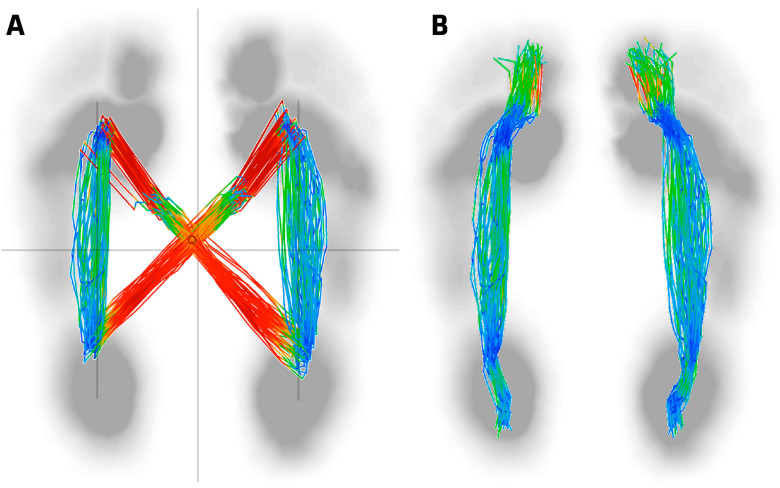
CoP analysis. (**A**) Butterfly analysis. (**B**) Left and right running line. Speed levels (red: fast, green: intermediate, blue: slow).

For the biomechanical analysis, the patient was asked to walk on the Zebris FDM platform (see Figure 3). The butterfly diagram was used [24,30] to examine the path of the center of pressure (CoP) during specific step cycles. This diagram, which presents the CoP tracks in different colors corresponding to speed levels (see Figure 4), provides a useful visual representation to understand the distribution of body weight during gait. In addition, both the anteroposterior and lateral displacement of the CoP were evaluated to analyze the dynamics of the movement. This detailed approach using the Zebris platform provides essential information on postural stability and CoP dynamics, which facilitates a comprehensive gait assessment and the identification of abnormal patterns.

As for the anteroposterior displacement (mm), we evaluated the forward or backward movement of the CoP intersection point along all steps. In this measure, we consider the displacement toward an anterior position positive, and negative when the CoP moves toward a posterior position.

Regarding lateral displacement (mm), we analyzed the left-to-right movement of the CoP intersection point during the pitch cycles. We associate a positive value when the CoP is located on the right side and a negative value when it is positioned on the left side.

This detailed approach using the Zebris platform provides us with essential information on weight distribution, postural stability, and CoP dynamics, allowing for a comprehensive assessment of gait and facilitating the identification of abnormal patterns that may require specific therapeutic interventions.

### 2.5. Variables

To reduce potential bias, this study used standardized radiographic measurements to classify occlusal relationships and an independent examiner performed all statistical analyses without knowledge of group assignment.

Variable Butterfly diagram
Length of Line of March

The parameter known as “Length of Line of March” refers to the position of the focal pressure point (FPP). Only the ground contacts on one side of the body are considered. This characteristic encompasses the FPP advance on all recorded steps on one side of the body.

Half Support Phase

This parameter denotes the average extent of the lines showing the progression of the FPP of one side of the body, taking into account all ground contacts.

Anteroposterior Position

This metric clarifies the anterior or posterior displacement of the FPP intersection point in a chronological sequence within the cyclogram display, considering all steps. The starting point or zero is the most posterior position where the heel makes contact with the ground.

Lateral Displacement

This indicator delineates the left/right displacement of the FPP intersection point in a chronological sequence within the cyclogram display, encompassing all steps. A negative value denotes a shift to the left, whereas a positive value indicates a shift to the right.

The starting position or zero is represented as the center point of the display.

Gait analysis variables

In the analysis of gait parameters, several variables are examined that provide detailed information about the walking process. These include geometric measurements, time phases, and force characteristics that reveal key aspects of human locomotion.

Foot rotation, expressed in degrees, describes the angle between the longitudinal axis of the foot and the direction of motion. A negative value indicates inward rotation, whereas a positive value indicates outward rotation. The step width, measured in centimeters, indicates the distance between the right and left foot during gait. On the other hand, step length, also in centimeters, represents the distance between the heel contact on one side of the body and the heel contact on the opposite side.

The step time, expressed in seconds, corresponds to the phase within a gait cycle between heel contact on one side of the body and heel contact on the opposite side. The stance phase, measured in percent, describes the period of a gait cycle in which the foot has contact with the ground. This period is divided into subphases, such as the loading response phase, which spans from initial ground contact to toe-off of the contralateral toe, and the mid-stance phase, which involves toe-off of the contralateral toe and transfer of the body’s center of gravity onto the weight-bearing foot.

The swing phase, also measured in percent, refers to the period of a gait cycle in which the foot has no contact with the ground. This includes subphases such as the pre-swing phase, which begins at initial contact on the opposite side and ends at toe-off on the observed side of the body, and the swing phase, which is the period during which the foot is in the air.

In addition to these geometric and temporal measurements, force aspects, such as average force and peak pressure, are considered and plotted throughout the gait cycle. These parameters provide information on the load distribution and forces acting during gait, which is crucial for understanding the biomechanics and efficiency of human movement.

### 2.6. Statistical Analysis

An observer outside the experimental setup performed all analyses. Mean and standard deviation (SD) were used to express the data. The Kolmogorov–Smirnov test was used to evaluate the assumption of normality. Levene’s test was also used to calculate the assumption of homogeneity of variance. The significance level was set at *p* > 0.05. SPSS 24 (SPSS 24 Inc., Chicago, IL, USA) and Jeffreys’s Amazing Statistical Package (JASP V0.16.4, Amsterdam, The Netherlands) were used for statistical analysis and graphical representation of the data, respectively. To determine whether anthropometric characteristics between groups were homogeneous (*p* > 0.05), a one-way *t*-test was used to examine the data. The difference between the groups with and without dental pathology was performed using the *t*-test for independent samples (Student’s *t*-test). In this analysis, the groups were used as independent variables. The ES was calculated by determining Cohen’s d coefficient, which was then expressed as the standardized mean change difference. The SE was classified as trivial (<0.20), small (0.20–0.59), moderate (0.60–1.19), large (1.20–1.99), or very large (>2.00) [38].

## 3. Results

### 3.1. Participation Flow and Sample Characteristics

A total of 29 subjects were enrolled to participate in this study. Upon examination of baseline data, no significant differences between groups were observed across any parameter, as outlined in Table 1.

### 3.2. Results and Main Outcomes of Butterfly Diagram

Firstly, concerning the length of the marching line, there were no statistically significant differences observed in either the left (*p* = 0.14) or right (*p* = 0.20) sides between the group with no pathology (217.21 ± 30.49 mm and 213.15 ± 32.72 mm, respectively) and the group with dental pathology (198.76 ± 34.28 mm and 196.53 ± 35.08 mm, respectively). Effect sizes, measured by Cohen’s d, were moderate (0.57 and 0.49 for the left and right sides, respectively) (see Table 2).

**Figure 5:**
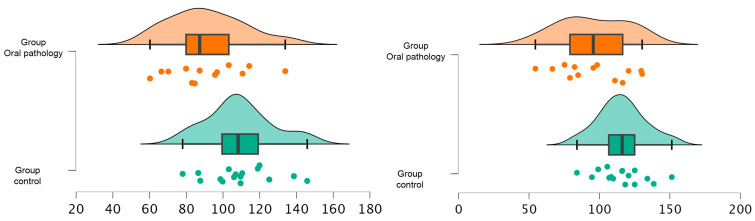
The figure shows the differences produced in main outcomes per group. Statistically significant differences between groups.

However, significant differences were noted in the medio-lateral position of the marching line. The group with no pathology exhibited a significantly greater medio-lateral position compared to the group with dental pathology, both on the left (*p* = 0.01) and right (*p* = 0.02) sides (115.85 ± 16.98 mm vs. 95.74 ± 24.47 mm for the left side and 109.03 ± 18.03 mm vs. 91.23 ± 20.80 mm for the right side). The effect sizes for these differences were large, measuring 0.97 and 0.92 for the left and right sides, respectively (see Figure 5).

No significant differences were observed between the two groups in terms of anterior/posterior position (*p* = 0.38), with mean values of 2.76 ± 3.67 mm for the group with no pathology and 1.44 ± 4.17 mm for the group with dental pathology. Likewise, there were no significant differences in lateral displacement (*p* = 0.78), with mean values of 1.68 ± 4.70 mm for the group with no pathology and 1.44 ± 4.17 mm for the group with dental pathology. Cohen’s d values for these parameters suggested small effect sizes (−0.11 and 0.34, respectively).

### 3.3. Results and Main Outcomes of Gait Analysis

In the non-pathology group, the mean left phase line position was 115.85 ± 16.98 mm, whereas in the control group, it was 95.74 ± 24.47 mm. For the right gait, values of 109.03 ± 18.03 mm and 91.23 ± 20.80 mm were recorded, respectively (See Table 3). These differences are more pronounced and show a large effect size according to Cohen’s d (0.97 and 0.92, respectively), suggesting that the presence of medial pathology has a significant and considerable impact on the medial position of the phase line during gait.

As for the force exerted on the forefoot and heel during gait, significant and large magnitude differences were observed. For example, in the left forefoot, the non-pathology group exerted a force of 710.12 ± 165.21 N, whereas in the control group, it was 485.57 ± 199.32 N, with a Cohen’s d of 1.24, indicating a large size effect. Similarly, in the left heel, values of 548.89 ± 127.50 N and 360.15 ± 125.78 N were recorded, respectively, with a Cohen’s d of 1.49, indicating a very large size effect (see Table 3).

These differences in gait parameters suggest a significant alteration in foot biomechanics in the group with a pathology compared to the healthy group. These findings may have important implications for the oral health of the patients.

## 4. Discussion

The primary aim of our research was to investigate the relationship between body posture and dental occlusion, a topic of increasing significance in the dental community [14,39,40]. This interdisciplinary field not only deepens our understanding of how dental structure interacts with the biomechanics of the human body but also presents new avenues for diagnosing and treating various dental and postural conditions.

Situated within this context, our study sought to explore and analyze the association between body posture and dental occlusion. Through a comprehensive approach, we conducted detailed evaluations of body posture in motion alongside analyses of gait and occlusion in a representative patient sample.

Our findings unveiled notable differences between patients with skeletal pathologies and those with normal skeletal structures. These distinctions may stem from excessive traction of the musculature during the unipodal forward stance phase. Previous studies have also identified a correlation between skeletal class and foot conditions, such as flat foot, pes cavus, and normal foot [41]. Unlike prior research, which typically focused on tooth structure and foot shape, our study pioneered an examination of the interaction between dynamic body posture, gait, and dental occlusion.

These results suggest that assessing musculature could serve as a pivotal entry point for designing targeted therapeutic interventions [42,43] and conducting comprehensive examinations in patients undergoing dental treatments. Continued research is imperative for establishing robust correlations between the analyzed variables. Furthermore, understanding the biomechanical dynamics of the human body has significant implications for physical performance and health, particularly in the realm of oral and dental studies.

In addition to the above, we aimed to correlate known rachis positions affecting balance and plantar pressures [44] with skeletal class and occlusal pathologies. Prior studies have indicated that cervical posture correlates with skeletal class [45,46], and there’s evidence linking temporomandibular disorders with postural changes and abnormal plantar pressure distributions [47]. Our study, utilizing quantitative data through Zebris FDM, a validated measuring instrument for gait and plantar pressure analysis, found significant differences between groups.

Another crucial aspect of our study was analyzing center of pressure (CoP) dynamics [4] and the potential association with skeletal class. Although no significant differences were found in anteroposterior CoP displacement among skeletal classes, similar to previous findings [13,48,49], our results suggest that jaw biomechanics could influence body weight distribution, subsequently affecting posture and balance. Significant differences in maximum heel forces were found with a large effect size relative to non-pathological cases, with existing studies linking lower limb differences to plantar pressures [50]. Moreover, foot function affects cervical function [51], just as walking in heels and anteriorizing plantar pressures modify spine curvature and gait velocity [52]. Our study also highlighted statistically significant differences in step length between groups, with those without dental pathology exhibiting greater mean step lengths. This study represents the first attempt to quantitatively relate Zebris FDM data with pathological skeletal classes and plantar pressures.

### Limitations of this Study

This study, although it provides a first approximation to the possible connection between body posture and dental occlusion, has several limitations that should be taken into account. Firstly, the sample size was relatively small (n = 29), which limits both the statistical power and the ability to generalize the results to larger populations. Furthermore, due to the cross-sectional design of this study, it is not possible to establish causal relationships between the variables analyzed. Further longitudinal and experimental studies would be necessary to confirm the existence of direct effects of posture on occlusion. In addition, the assessment of posture was mainly focused on the analysis of the center of pressures, excluding other relevant body regions. For future research, it would be important to incorporate more comprehensive assessments covering different body segments and planes. Finally, possible confounding variables, such as the presence of cervical pain or low back pain, which could influence the relationship between occlusion and posture, were not taken into account.

## 5. Conclusions

In conclusion, our study highlights the need for more extensive and detailed research to better understand the complex interrelationships between body posture and dental occlusion. Studies with larger samples and more precise assessment methods are needed to confirm and extend our findings. However, these preliminary results underscore the importance of considering both body posture and dental occlusion in the diagnosis and treatment of dental patients. This integrated perspective may lead to better clinical outcomes and more holistic patient care.

## Figures and Tables

**Table 1:** Descriptions and *t*-tests of general group descriptions per group.

Variable	All Participants (n = 29)	Group Control Non-Pathologic (n = 13)	Group Pathology (n = 16)	*p*-Value
Age	21.73 ± 8.10	21.23 ± 10.34	22.12 ± 6.20	0.77
Height cm	165.00 ± 13.21	162.38 ± 14.10	167 ± 12.55	0.35
Weight kg	64.74 ± 14.36	61.69 ± 16.29	67.06 ± 12.72	0.32
Body mass index, kg/m^2^	23.36 ± 2.08	22.88 ± 2.47	23.73 ± 1.71	0.27

**Table 2:** Data butterfly diagram.

	Group Control Non-Pathology (SD)	Group Dental Pathology (SD)	*p*-Value	Cohen’s d
Running line length I, mm	217.21 ± 30.49	198.76 ± 34.28	0.14	0.57
Running line length D, mm	213.15 ± 32.72	196.53 ± 35.08	0.20	0.49
Phase line of mean position I, mm	115.85 ± 16.98	95.74 ± 24.47	0.01 *	0.97
Mean position phase line D, mm	109.03 ± 18.03	91.23 ± 20.80	0.02 *	0.92
Ant./post. position, mm	2.76 ± 3.67	1.44 ± 4.17	0.38	0.34
Lateral displacement, mm	1.68 ± 4.70	2.23 ± 5.77	0.78	−0.11
Max gait line velocity, cm/s	244.88 ± 56.50	247.87 ± 95.84	0.92	−0.04

* *p* < 0.05.

**Table 3:** Descriptive data (means, ±SD) between groups on gait study variables.

		Group Control Non-Pathology (SD)	Group Dental Pathology (SD)	*p*-Value	Cohen’s d
GAIT PHASES	Forefoot left, N	710.12 ± 165.21	485.57 ± 199.32	2.59 × 10^−3^	1.24
	Forefoot right, N	717.70 ± 171.85	486.52 ± 198.39	2.32 × 10^−3^	1.26
	Heel to the left, N.	548.89 ± 127.50	360.15 ± 125.78	<0.001 *	1.49
	Heel to the right, N.	535.71 ± 131.57	342.70 ± 108.40	<0.001 *	1.58
	Forefoot left, %	104.83 ± 7.02	93.76 ± 8.63	<0.001 *	1.42
	Forefoot right, %	105.87 ± 6.08	93.99 ± 8.50	<0.001 *	1.64
	Heel left, %	81.43 ± 8.90	71.48 ± 6.52	2.33 × 10^−3^	1.25
	Heel right, %	79.55 ± 10.49	68.65 ± 6.52	2.98 × 10^−3^	1.22
GEOMETRY	Foot rotation I, grade	7.16 ± 4.57	3.78 ± 7.63	0.15	0.55
	Foot rotation D, grade	8.74 ± 4.48	5.26 ± 6.42	0.10	0.64
	Step length I, cm	58.39 ± 9.41	41.08 ± 10.12	<0.001 *	1.78
	Step length D, cm	58.36 ± 9.96	42.18 ± 9.48	<0.001 *	1.66
	Double step length, cm	116.75 ± 19.28	83.25 ± 19.46	<0.001 *	1.73
	Step extension, cm	11.18 ± 2.35	12.06 ± 2.57	0.34	−0.36
THREE FOOT ZONE ANALYSIS	Maximum force 1 I, N	723.09 ± 167.97	516.25 ± 174.21	3.13 × 10^−3^	1.21
	Maximum force 1 D, N	727.54 ± 182.09	523.90 ± 195.14	7.32 × 10^−3^	1.08
	Moment of maximum force 1 (t1) I, %	17.19 ± 2.79	19.85 ± 3.63	0.03 *	−0.83
	Moment of maximum force 1 (t1) D, %	17.38 ± 3.81	19.77 ± 3.09	0.08	−0.68
	Maximum force 2 I, N	705.86 ± 166.43	499.97 ± 180.92	3.62 × 10^−3^	1.19
	Maximum force 2 D, N	710.99 ± 169.98	479.89 ± 175.84	1.66 × 10^−3^	1.34
MAXIMUM FORCE, N	Maximum force forefoot I, N	688.10 ± 156.79	466.90 ± 197.32	2.30 × 10^−3^	1.26
	Maximum force forefoot D, N	696.52 ± 163.65	466.58 ± 193.59	1.77 × 10^−3^	1.29
	Maximum force midfoot I, N	132.41 ± 72.18	103.91 ± 59.75	0.26	0.43
	Maximum force midfoot D, N	153.32 ± 98.17	109.77 ± 57.88	0.17	0.53
	Maximum force heel I, N	493.71 ± 118.78	318.38 ± 116.15	<0.001 *	1.49
	Maximum force heel D, N	470.62 ± 110.98	299.44 ± 97.82	<0.001 *	1.63
MAXIMUM PRESSURE	Maximum pressure forefoot I, N/cm^2^	38.73 ± 8.77	26.00 ± 10.44	1.36 × 10^−3^	1.33
	Maximum pressure forefoot D, N/cm^2^	37.78 ± 8.17	25.61 ± 11.04	2.04 × 10^−3^	1.27
	Maximum pressure midfoot I, N/cm^2^	14.84 ± 4.57	9.65 ± 2.14	<0.001 *	1.40
	Maximum pressure midfoot D, N/cm^2^	14.83 ± 4.30	10.11 ± 2.90	2.23 × 10^−3^	1.26
	Maximum pressure heel I, N/cm^2^	31.67 ± 7.59	25.52 ± 8.72	0.05	0.76
	Maximum pressure heel D, N/cm^2^	29.64 ± 5.18	22.41 ± 5.05	<0.001 *	1.41

Note: contrast of student. * Brown–Forsythe contrast is significant (*p* < 0.05), suggesting non-compliance with the assumption of equality of variances.

## Data Availability

The data presented in this study are available upon request to the corresponding author.

## Supplementary Materials

The following supporting information can be downloaded at: https://www.mdpi.com/article/10.3390/s24061921/s1, Table S1: STROBE checklist.
